# Effects of interleukin‐17A in nucleus pulposus cells and its small‐molecule inhibitors for intervertebral disc disease

**DOI:** 10.1111/jcmm.13828

**Published:** 2018-09-11

**Authors:** Kaori Suyama, Daisuke Sakai, Noriaki Hirayama, Yoshihiko Nakamura, Erika Matsushita, Hayato Terayama, Ning Qu, Osamu Tanaka, Kou Sakabe, Masahiko Watanabe

**Affiliations:** ^1^ Department of Anatomy and Cellular biology Basic Medical Science Tokai University School of Medicine Kanagawa Japan; ^2^ Department of Orthopaedic Surgery Surgical Science Tokai University School of Medicine Kanagawa Japan; ^3^ Institute of Advanced Biosciences Tokai University Kanagawa Japan

**Keywords:** interleukin ‐17A, intervertebral disc, nucleus pulposus, small‐molecule inhibitor

## Abstract

Intervertebral discs (IVD) degeneration, which is caused by ageing or mechanical stress, leads to IVD disease, including back pain and sciatica. The cytokine interleukin (IL)‐17A is elevated in NP cells during IVD disease. Here we explored the pharmacotherapeutic potential of IL‐17A for the treatment of IVD disease using small‐molecule inhibitors that block binding of IL‐17A to the IL‐17A receptor (IL‐17RA). Treatment of NP cells with IL‐17A increased expression of cyclooxygenase‐2 (COX‐2), IL‐6, matrix metalloproteinase (MMP)‐3 and MMP‐13. These increases were suppressed by an IL‐17A‐neutralizing antibody, and small molecules that were identified as inhibitors by binding to the IL‐17A‐binding region of IL‐17RA. IL‐17A signalling also altered sulphated glycosaminoglycan deposition and spheroid colony formation, while treatment with small‐molecule inhibitors of IL‐17A attenuated this response. Furthermore, mitogen‐activated protein kinase pathways were activated by IL‐17A stimulation and induced IL‐6 and COX‐2 expression, while small‐molecule inhibitors of IL‐17A suppressed their expression. Taken together, these results show that IL‐17A is a valid target for IVD disease therapy and that small‐molecule inhibitors that inhibit the IL‐17A–IL‐17RA interaction may be useful for pharmacotherapy of IVD disease.

## 1 INTRODUCTION

Intervertebral disc (IVD) degeneration, which results from ageing or mechanical stress, is clinically related to disc herniation, spinal canal stenosis and spinal deformities.^1^ These disorders cause lower back pain and sciatica, which can have a profound effect on a patient's quality of life and cause considerable socio‐economic burden.^2^ The IVD is an avascular organ, consisting of an outer fibrocartilaginous annulus fibrosus (AF), which surrounds a gel‐like nucleus pulposus (NP) that acts as a shock absorber and maintains backbone mobility.^3^ The NP is comprised mainly of water, but also collagen fibrils, various proteoglycans for shock absorption and cells of the NP, which are adapted to survive in this hypoxic environment.^4^, ^5^, ^6^ In degenerated IVD tissue, various inflammatory factors including interleukin (IL)‐α/β, IL‐2, IL‐4, IL‐6, IL‐8, IL‐10, nitric oxide, tumour necrosis factor α (TNF‐α), matrix metalloproteinases (MMPs) and prostaglandin E_2_ (PGE2) increase in the NP and stimulate‐specific intracellular signalling pathways that further enhance this degenerative process^7^, ^8^, ^9^, ^10^, ^11^, ^12^ through, for example, the degradation of collagen fibres and hydrophilic proteoglycans within the extracellular matrix(ECM).^13^ PGE2 is synthesized by two cyclooxygenase (COX) isoforms, COX‐1 and COX‐2.^14^ COX‐2 expression is induced in response to inflammatory stimuli^15^ and mechanical stress, and its expression initiates the degenerative cascade.^16^ COX‐2 and PGE‐2 play key roles in lower back pain and sciatica.^7^, ^8^, ^17^ As such, COX‐2 is considered an important mediator of the IVD degenerative process^8^ and is a therapeutic target for IVD disease.^8^

Recently, IL‐17 was reported to be increased in NP cells of degenerated or herniated IVDs, along with IL‐4, IL‐6, IL‐12 and interferon‐γ. IL‐17 is a cytokine produced by the T helper 17 subset of CD4^+^ T cells and plays critical roles in various inflammatory disorders.^18^, ^19^ Its family is comprised of six members: IL‐17A to IL‐17F.^20^ IL‐17A binds to its cognate receptor (IL‐17RA) and activates many intracellular signaling factors, including those of the nuclear factor kappa‐light‐chain‐enhancer of activated B cells or mitogen‐activated protein kinase (MAPK) pathways.^21^ IL‐17A also synergistically increases the release of inflammatory mediators in disc cells.^22^, ^23^ With respect to the relationship between IL‐17A and other cytokines, IL‐6 is one of a range of factors triggered when CD4^+^ T cells undergo differentiation into Th17 cells, which produce IL‐17 in response to IL‐6 and transforming growth factor β via Retinoic acid receptor‐related Orphan Receptor (ROR)‐ γ.^24^, ^25^ IL‐17A also induces expression of IL‐6 in fibroblasts^26^ and triggers a positive feedback loop of IL‐6 signalling.^27^ In IVD disease and degeneration, IL‐6 can potentiate the catabolic actions of other cytokines, including IL‐1 and TNF‐α,^28^ induce apoptosis^29^ and cause loss of IVD matrix proteins including MMP family members, such as MMP‐3 or MMP‐13.^28^, ^30^ In addition, IL‐6 and IL‐6 receptor expression levels are responsive to IVD injury.^31^ Thus, IL‐6 plays an important role in the degenerative progression of NP cells and the various symptoms associated with degenerative disorders. Additionally, IL‐17A may lead to an increase in COX‐2 expression via the activation of MAPK pathways in disc degeneration.^32^

Therefore, IL‐17A may offer a potential target for therapeutic intervention for disc degeneration. However, there are few studies examining the role of IL‐17 in disc cells. To address this, we focused on the crosstalk between IL‐17A, IL‐6 and COX‐2 and investigated the effect of IL‐17A on NP cells under hypoxic conditions (1% O_2_), reflecting the in vivo context. The main objectives of this study were to examine the pharmacotherapeutic potential of IL‐17A for the treatment of degenerative discs. We analysed the structure of IL‐17A and the IL‐17A receptor (IL‐17RA) and identified small‐molecule inhibitors that bind to the IL‐17A–IL‐17RA docking region and inhibit IL‐17A signalling in NP cells. We evaluated the effects of these inhibitors as a potential new therapeutic strategy for disc diseases.

## 2 MATERIALS AND METHODS

### 2.1 Human samples and histology

Informed consent was obtained from each patient for the use of their IVD tissue according to the Declaration of Helsinki. Ethical approval was obtained from the Institutional Ethics Review Board of Tokai University School of Medicine. A total of 11 IVD tissues were dissected from six patients with lumbar herniation and five patients under 16 years of age with idiopathic scoliosis. The samples were evaluated according to Pfirrmann's magnetic resonance classification,^33^ and grades III, IV and V were considered to be degenerated IVD and grades I and II considered to be non‐generated IVD. Using this classification, IVDs from all six lumbar herniated disc patients were considered degenerated while those from all five idiopathic scoliosis patients were non‐degenerated. The samples were fixed in 4% paraformaldehyde in phosphate‐buffered saline (PBS) and embedded in paraffin. The sections were deparaffinized in xylene, rehydrated through graded ethanol and incubated with IL‐17A antibody (#bs‐2140R; Bios) diluted in 1% bovine serum albumin in PBS overnight at 4°C. Samples were then stained with horseradish peroxidase (HRP)‐conjugated goat anti‐rabbit IgG (Sigma‐Aldrich, St. Louis, MO, USA). Staining was visualized using diaminobenzidine (Nakarai Tesque, Kyoto, Japan). Nuclei were stained with haematoxylin. All specimens were viewed under a microscope (IX70; Olympus, Tokyo, Japan). Positively immunolabelled cells were calculated as a percentage of the total number of cells per high‐power field in each section.

### 2.2 Isolation of NP cells, cell treatments and hypoxic culture conditions

Rat NP cells were isolated from male Sprague‐Dawley rats (11 weeks old) using a modified method previously reported by Risbud et al.^5^ Briefly, IVDs from the lumbar and coccygeal regions were dissected from rats under deep anaesthesia using aseptic conditions. The gel‐like NP was separated from the AF, and the NP tissue was minced by pipetting. The isolated cells were maintained in Dulbecco's modified Eagle's medium (DMEM) (Nakarai Tesque) supplemented with 10% foetal bovine serum (FBS; Gibco, Grand Island, NY, USA) and antibiotics and cultured in a Hypoxia Chamber (MIC‐101; Billups Rothenberg Inc., Del Mar, CA, USA) containing 1% O_2_, 5% CO_2_ and 94% N_2_. Human NP cells were digested with TrypLE Express (Gibco) and then collagenase‐P (Roche, Indianapolis, IN, USA), washed twice with a‐minimal essential medium (Gibco) and cultured in DMEM (Nakarai Tesque) supplemented with 10% FBS and antibiotics. They were cultured under similar conditions as rat NP cells.

### 2.3 Treatment with IL‐17A, Anti‐IL‐17A and MAPK inhibitors

Rat NP cells were treated with 20‐50 ng/mL IL‐17A (PeproTech Inc., Rocky Hill, NJ, USA) for 1‐24 hours. To inhibit MAPKs, NP cells were pre‐treated with 10 μmol/L p38 inhibitor (SB203580; AdipoGen, Incheon, Korea), ERK inhibitor (PD98059; Cayman Chemical, Ann Arbor, MI, USA) or JNK inhibitor (SP600125; Cayman Chemical) for 1 hours and then treated with 50 ng IL‐17A. For IL‐17A neutralization assays, 0.5 μg/mL of anti‐IL‐17A‐neutralizing antibody (#DDX0336P‐50; Novus, St. Louis, MO, USA) was mixed with 50 ng/mL of IL‐17A for 1 hour in medium and then added to the NP cells.

### 2.4 Analysis of IL‐17A–IL‐17RA structure and identification of small‐molecule inhibitors

Virtual screening of small‐molecule inhibitors was undertaken based on the crystal structure of a complex between IL‐17A and IL‐17RA.^20^ The possible binding site of small molecules on the surface of IL‐17RA where IL‐17A is deeply bound was identified using the alpha site finder function implemented in Molecular Operating Environment (Chemical Computing Group, Montreal, QC, Canada). The docking simulations, by use of ASEDock,^34^ were performed between the site and a set of 1099 drug molecules used clinically in Japan. The molecular weights of the drugs ranged between 120 and 750 Da. The docking score of GBVI/WSA_dG,^35^ which expresses the protein ligand‐binding free energy, was employed to judge the binding affinity between the receptor and small molecules. The molecular characteristics of 55 molecules with the lowest GBVI/WSA_dG values were calculated using DRFF software^36^ to make a filter function to select molecules with similar molecular characteristics. Using this filter function, 5413 compounds were selected from a chemical database of nearly six million small molecules. The docking simulations between these 5413 compounds and IL‐17RA were undertaken using ASEDock. The four compounds with the lowest GBVI/WSA_dG values were used in in vitro experiments. Among them, STK630921 (STK) showed a significant effect in suppressing IL‐17RA activity, revealing its strong binding affinity to IL‐17RA.

### 2.5 Real‐time RT‐PCR analysis

Total RNA was extracted from NP cells using RNAeasy mini columns (Qiagen, Hilden, Germany). Before elution from the column, RNA was treated with RNase‐free DNase I (Qiagen). The purified, DNA‐free RNA was converted to cDNA using High‐Capacity cDNA Reverse Transcription Kits (Applied Biosystems, Foster City, CA, USA). Template cDNA and gene‐specific primers were added to Power SYBR Green Master Mix (Applied Biosystems), and mRNA expression was quantified using the Step One Plus Real‐time PCR System (Applied Biosystems). β‐actin was used to normalize gene expression. Melting curves were analysed to verify the specificity of the RT‐PCR and the absence of primer dimer formation.

### 2.6 Plasmids

The COX‐2 promoter luciferase constructs phPES2‐1432/+59 were kindly provided by Dr. Akihiko Hiyama (Tokai University, Kanagawa, Japan).^37^ We used the vector pGL4.74 (Promega, Madison, WI, USA) containing the *Renilla reniformis* luciferase gene as an internal transfection control.

### 2.7 Transfections and dual‐luciferase assay

Cells were transferred to 96‐well plates (8 × 10^3^ cells/well) 24 hours before transfection. Cells were transfected with phPES2‐1432/+59 or empty backbone plasmids and pGL4.74. Lipofectamine 2000 (Invitrogen, Carlsbad, CA, USA) was used as the transfection reagent. The reporter activities were measured after culturing under hypoxic conditions for 24 hours. The Dual‐Luciferase Reporter Assay system (Promega) was used for measurements of firefly and *Renilla luciferase* activities using a luminometer (TD‐20/20; Turner Designs, Fresno, CA, USA).

### 2.8 Protein extraction, western blotting and immunoprecipitation

At the indicated time‐points after treatment, cells were placed on ice and then washed with ice‐cold PBS. To prepare total cellular proteins, cells were lysed with lysis buffer containing 10 mmol/L Tris–HCl, pH 7.6, 50 mmol/L NaCl, 5 mmol/L EDTA, 1% Nonidet P‐40, complete protease inhibitor cocktail (Roche), 1 mmol/L NaF and 1 mmol/L Na_3_VO_4_. Proteins were fractionated by sodium dodecyl sulphate‐polyacrylamide gel electrophoresis and transferred to Immobilon‐P polyvinylidene difluoride membranes (Millipore, Billerica, MA, USA). The membranes were blocked with blocking buffer (5% BSA, 0.1% NaN_3_ in PBS) and then incubated overnight at 4°C with antibodies against IL‐6 (#bs‐0782R; Bios), COX‐2 (#NB100‐689SS; Novus), p38 (#8690; Cell Signaling Technology, Danvers, MA, USA), phosphorylated p38 (#4511; Cell Signaling Technology), ERK (#4695; Cell Signaling Technology), phosphorylated ERK (#4370; Cell Signaling Technology), JNK (#9252; Cell Signaling Technology), phosphorylated JNK (#AF1205; R&D Systems, Minneapolis, MN, USA) or β‐actin (#A2228; Sigma‐Aldrich). All antibodies were diluted in Can Get Signal Immunoreaction Enhancer Solution (Toyobo, Tokyo, Japan). Chemiluminescence signals were visualized with Immobilon Western Chemiluminescent HRP Substrate (Millipore) and scanned using an Ez‐Capture MG imaging system (ATTO, Tokyo, Japan). The Western blot data were quantified by densitometric scans of the films using computer software for Macintosh, CS analyzer (ATTO). Western blot data are presented as band intensities normalized to that of the loading control (β‐actin).

### 2.9 Alcian blue staining

Nucleus pulposus cells were cultured for 8 days under hypoxic conditions, with 50 ng/mL IL‐17A or 50 μg/mL of STK added every other day. Cells were washed with PBS, treated with 20% formaldehyde solution for 15 minutes and washed again with PBS. The cells were stained with 0.1% Alcian blue in 0.1 mol/L HCl (pH 1.0) overnight and washed in PBS. The Alcian blue‐stained cultures were extracted at room temperature using 6 mol/L guanidine hydrochloride. The optical density (OD) of the extracted dye was measured at 670 nm.

### 2.10 Colony‐forming assay

To assess spheroid colony formation, single‐cell suspensions of 1.0 × 10^3^ human NP cells were inoculated into 35‐mm‐diameter dishes and cultured in 1 mL of MethoCult H4230 methylcellulose medium (Stem Cell Technologies) and were treated with 10‐100 ng/mL of IL‐17A and 50‐200 μg/mL of STK for 10 days. Colonies (>10 cells) were counted using an inverted microscope.

### 2.11 Statistical analysis

All measurements were performed at least three times, and the data are presented as the mean ± standard deviations (SD). Differences between groups were analysed using Student's *t* test or one‐way analyses of variance. Dunnett's test was used as post hoc test. Significance was set at *P *<* *0.05.

## 3 RESULTS

### 3.1 Induction of IL‐17A expression in NP cells of human herniated discs

**Figure 1:**
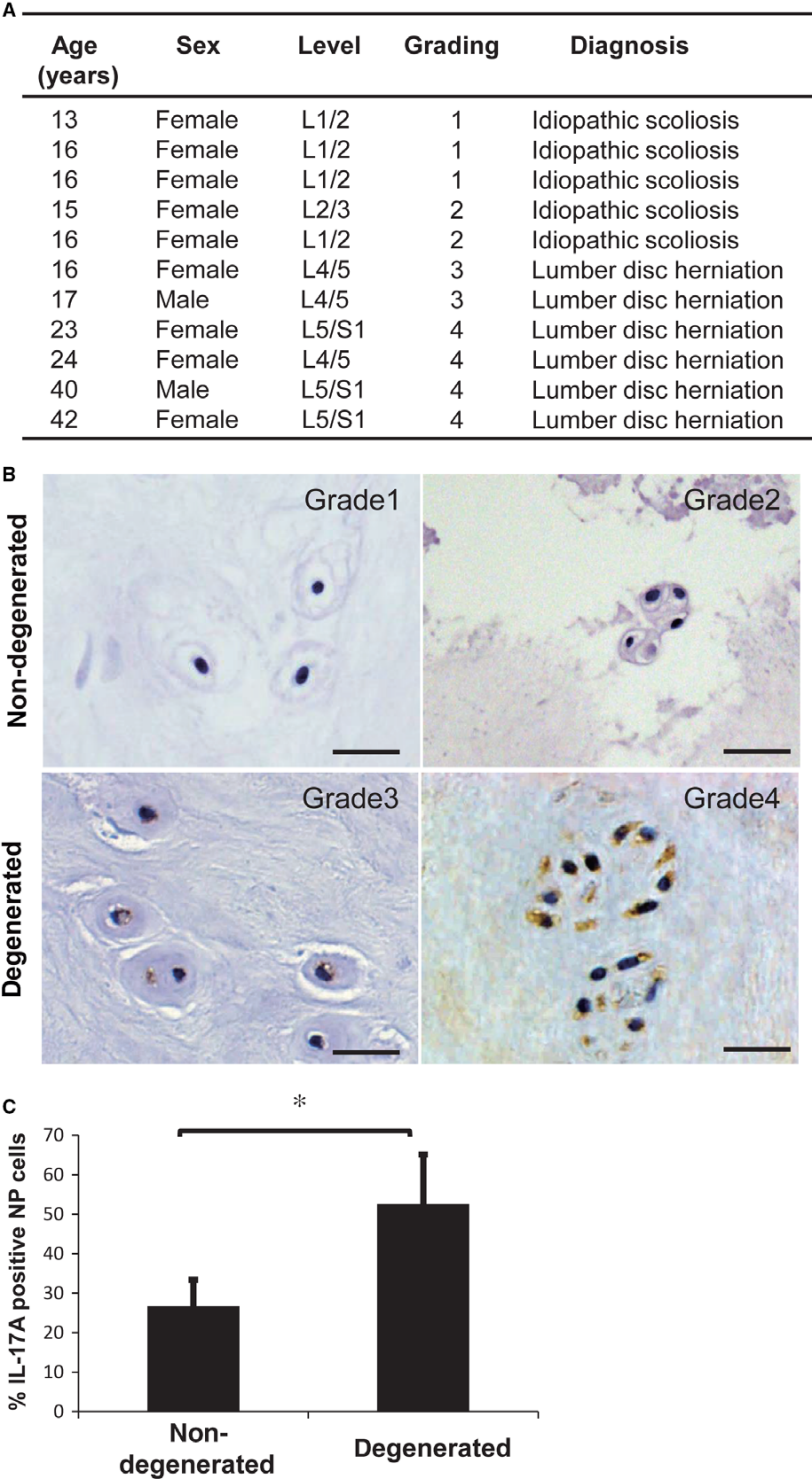
A, The list that was itemized the detailed conditions of patients who underwent surgery. B, Immunohistochemical staining of IL‐17A in nucleus pulposus (NP) cells of a non‐degenerated human intervertebral disc and a degenerative intervertebral disc. IL‐17A expression was prominent in the NP cells of herniated discs. Scale bars: 10 μm. C, The percentage of IL‐17A‐positive NP cells in human intervertebral discs. There was a significant increase in IL‐17A‐positive cells in the degenerative discs. Results shown as mean ± SD; n = 5‐6, *P <* *0.05

We first classified the level of degeneration in IVD samples according to Pfirrmann's magnetic resonance classification^33^ and considered IVD samples of grades III to V to be degenerative (Figure 1A). Among our samples, six herniated disc samples were observed from patients with grade III or IV IVD, whereas samples from the five patients with idiopathic scoliosis were non‐degenerated (Figure 1A,B).

To evaluate the involvement of IL‐17A in IVD degeneration, we measured expression of IL‐17A in NP cells in human IVD samples by immunohistochemistry. IL‐17A expression was prominent in the NP cells of herniated discs (Figure 1B), with a significantly higher percentage of IL‐17A‐positive cells compared with non‐degenerated IVD samples (Figure 1A‐C). This confirms others’ findings that IL‐17A is elevated in the NP cells of degenerative disc tissues.

### 3.2 IL‐17A treatment increases IL‐6 and COX‐2 expression and down‐regulates sulphated glycosaminoglycan staining in primary NP cells

**Figure 2:**
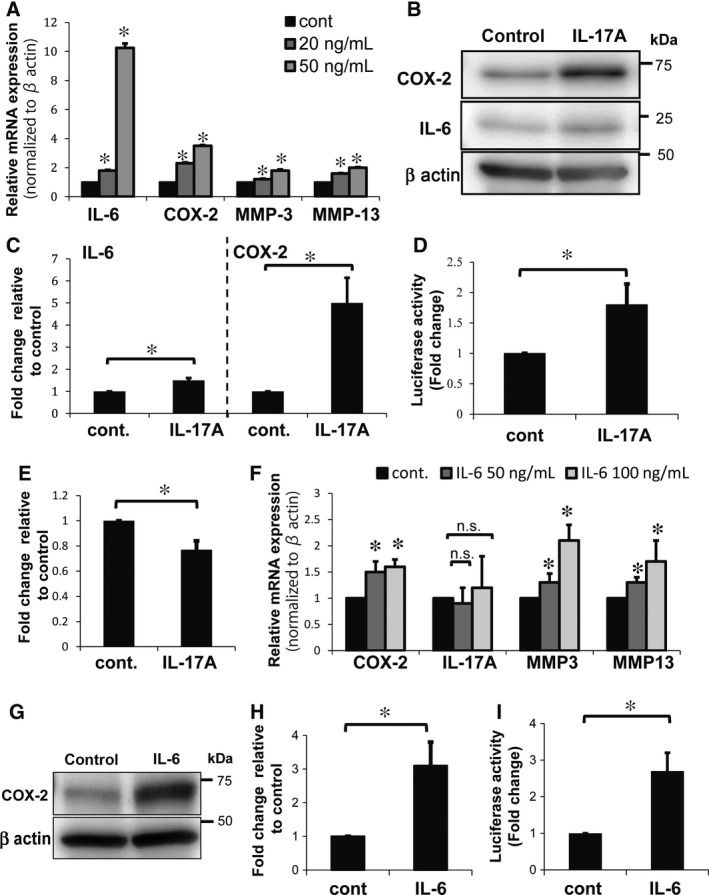
Effects of IL‐17A or IL‐6 treatment on NP cells under hypoxia. A, Real‐time PCR analysis of rat NP cells treated with 20‐50 ng/mL IL‐17A and cultured under hypoxia (1% O_2_). The control cells were not treated with IL‐17A. IL‐17A treatment significantly increased expression of IL‐6, COX‐2, MMP‐3 and MMP‐13 mRNA. Results shown as mean ± SD; n = 4, *P <* *0.05. B, C, Western blot analysis of NP cells treated with 50 ng/mL IL‐17A for 24 hours under hypoxic conditions. IL‐17A treatment led to a significant increase in expression of IL‐6 and COX‐2 proteins. Results shown as mean ± SD; n = 3, *P <* *0.05. D, Effects of IL‐17A on COX‐2 promoter activity in NP cells. Luciferase activity was assessed to show COX‐2 transcription in NP cells after treatment with 50 ng/mL of IL‐17A. IL‐17A treatment significantly increased COX‐2 promoter transcriptional activity. Results shown as mean ± SD; n =* *3, *P <* *0.05. E, Alcian blue staining quantifying of NP cells treated with 50 ng/mL IL‐17A. Optical density of the extracted dye was measured at 670 nm. Results shown as mean ± SD; n = 3, *P < 0.05. F, Real‐time PCR analysis of NP cells treated with 50‐100 ng/mL IL‐6 for 24 hours. The control cells were not treated with IL‐6. Treatment with IL‐6 led to significantly increased expression of COX‐2, MMP‐3 and MMP‐13 mRNA but had no effect on IL‐17A mRNA expression. Results shown as mean ± SD; n =* *3, *P <* *0.05. G, H, Western blot analysis of NP cells treated with 50 ng/mL IL‐6 for 24 hours under hypoxic conditions. IL‐6 treatment led to a significant increase in expression of COX‐2 proteins. Results shown as mean ± SD; n =* *3, *P <* *0.05. I, Effects of IL‐6 on COX‐2 promoter activity in NP cells. Luciferase activity was used to show COX‐2 promoter transcription in NP cells after treatment with 50 ng/mL of IL‐6, which significantly increased COX‐2 promoter transcription activity. Results shown as mean ± SD; n = 3, *P <* *0.05

Next, we assessed mRNA expression changes after IL‐17A stimulation (20 and 50 ng/mL for 24 hours) of rat NP cells under hypoxia (1% O_2_). IL‐17A treatment significantly increased IL‐6, COX‐2, MMP‐3, and MMP‐13 mRNA levels compared with untreated control cells (Figure 2A). We evaluated the protein levels of IL‐6, the mRNA of which was obviously elevated by IL‐17A treatment, and COX‐2, which is one of the major components of disc degeneration and which causes pain. With IL‐17A treatment, protein levels increased significantly, compared with untreated control cells (Figure 2B,C). To further confirm the effects of IL‐17A on COX‐2, we examined the transcriptional activity of the COX‐2 promoter. There was a significant increase in COX‐2 transcriptional activity in NP cells treated with 50 ng/mL IL‐17A for 24 hours under hypoxic conditions, compared with untreated control cells (Figure 2D). We also evaluated the effects of IL‐17A on the extracellular matrix (ECM) of NP cells. Alcian blue staining was used to detect the sulphated glycosaminoglycans of NP cells on day 8, and we observed that IL‐17A treatment lead to a significant decrease in sulphated glycosaminoglycans (Figure 2E).

### 3.3 IL‐6 increases COX‐2 expression in primary NP Cells

Next, because IL‐6 mRNA was prominently increased by IL‐17A stimulation, we evaluated the effects of IL‐6 in rat NP cells. Treatment with IL‐6 led to a significantly increased expression of COX‐2, MMP‐3 and MMP‐13 mRNA, like IL‐17A treatment, but had no effect on IL‐17A mRNA expression compared with untreated control cells (Figure 2F). In addition, we confirmed that COX‐2 protein expression (Figure 2G,H) and COX‐2 promoter transcriptional activity were significantly increased by IL‐6 treatment compared with untreated control cells (Figure 2I). These results suggest that IL‐6 induces expression of COX‐2 and MMPs, but not IL‐17A.

### 3.4 Anti‐IL‐17A antibody suppressed IL‐6, COX‐2 and MMP expression

**Figure 3:**
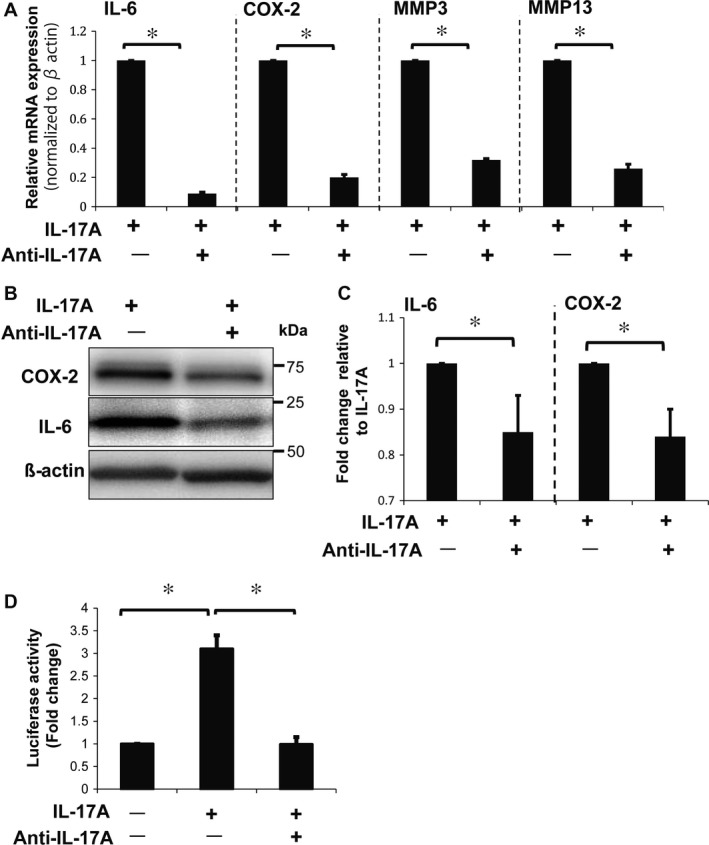
Effects of IL‐17A treatment with anti‐IL‐17A‐neutralizing antibody on NP cells. A, Real‐time PCR analysis of NP cells treated with 0.5 μg/mL of anti‐IL‐17A antibody and 50 ng/mL of IL‐17A for 24 hours showed a significant reduction in expression of IL‐6, COX‐2, MMP‐3 and MMP‐13 mRNA. B, C, Western blot analysis of NP cells with anti‐IL‐17A antibody treatment, confirming the reduction in expression of IL‐6 and COX‐2 protein. (D) COX‐2 promoter assay showing the loss of COX‐2 transcriptional activity after treatment with anti‐IL‐17A antibody and IL‐17A. Results shown as mean ± SD; n = 3, *P <* *0.05

To further evaluate the effects of IL‐17A on rat NP cells under hypoxic conditions, we inhibited the activity of IL‐17A using an IL‐17A‐neutralizing antibody. Treatment with IL‐17A and anti‐IL‐17A antibody caused a significant decrease in IL‐6, COX‐2, MMP‐3 and MMP‐13 mRNA expression levels compared with IL‐17A treatment alone (Figure 3A). Similarly, protein levels of IL‐6 and COX‐2, with treatment of IL‐17A and anti‐IL‐17A antibody, decreased significantly compared with IL‐17A treatment alone (Figure 3B,C). Additionally, the transcriptional activity of the COX‐2 promoter in NP cells treated with the anti‐IL‐17A antibody and IL‐17A was significantly decreased compared with its activity in cells treated with IL‐17A alone (Figure 3D). These results suggest that expression of both IL‐6 and COX‐2 can be inhibited by neutralizing the activity of IL‐17A.

### 3.5 Small‐molecule inhibitors bind to IL‐17RA and suppress the effect of IL‐17A on NP cells

**Figure 4:**
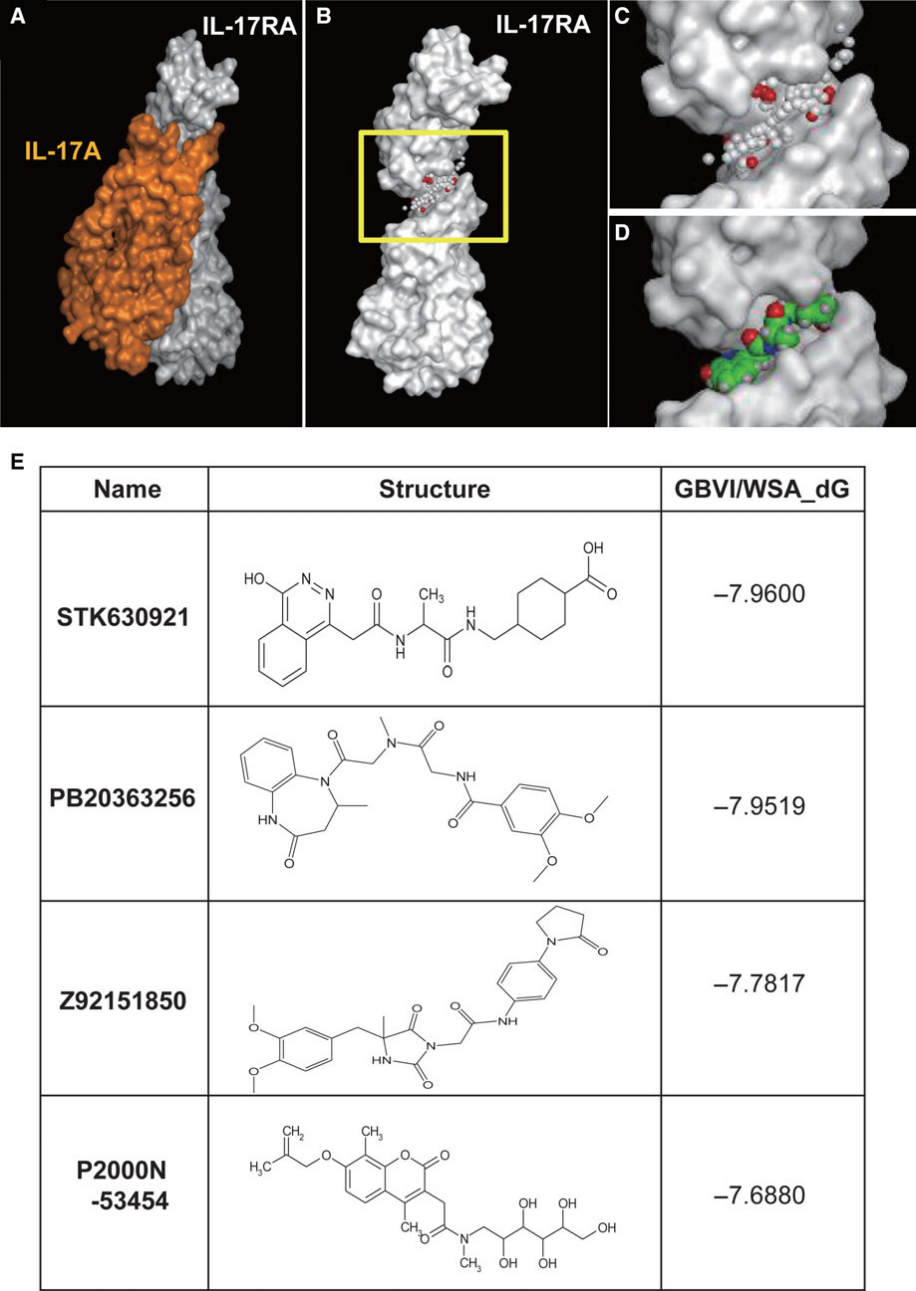
The structures of IL‐17A, IL‐17RA and small‐molecule inhibitors. A, X‐ray structure of IL‐17A bound to IL‐17RA. Molecular surface of a complex between IL‐17A (orange) and IL‐17RA (white). B, Molecular surface of IL‐17RA. The yellow boxed region is the binding site. C, The magnified view of the binding site (yellow boxed region in B). The cleft where a cluster of small spheres (alpha spheres) is localized represents the binding site for IL‐17A. White and red alpha spheres represent hydrophobic and hydrophilic pseudo atoms. D, Binding mode of STK630921 at the biding site of IL‐17A. STK630921 is depicted by a space‐filling model. Carbon, oxygen, nitrogen and hydrogen atoms are green, red, blue and white, respectively. E, Chemical structures of small‐molecule inhibitors for IL‐17A–IL‐17RA

We analysed the spatial structure of the protein–protein interactions between IL‐17A and IL‐17RA^20^, ^36^ (Figure 4A‐C) and identified four small‐molecule inhibitors (Figure 4D,E) that accessed the IL‐17A‐binding site region of IL‐17RA, as determined by in silico analysis of numerous drug molecules used clinically in Japan.^34^, ^35^, ^38^ The binding site, represented by a cluster of small spheres, is shown in Figure 4B,C. The mode of STK630921 (STK) binding to IL‐17RA and the chemical structure of the four compounds are shown in Figure 4D,E.

**Figure 5:**
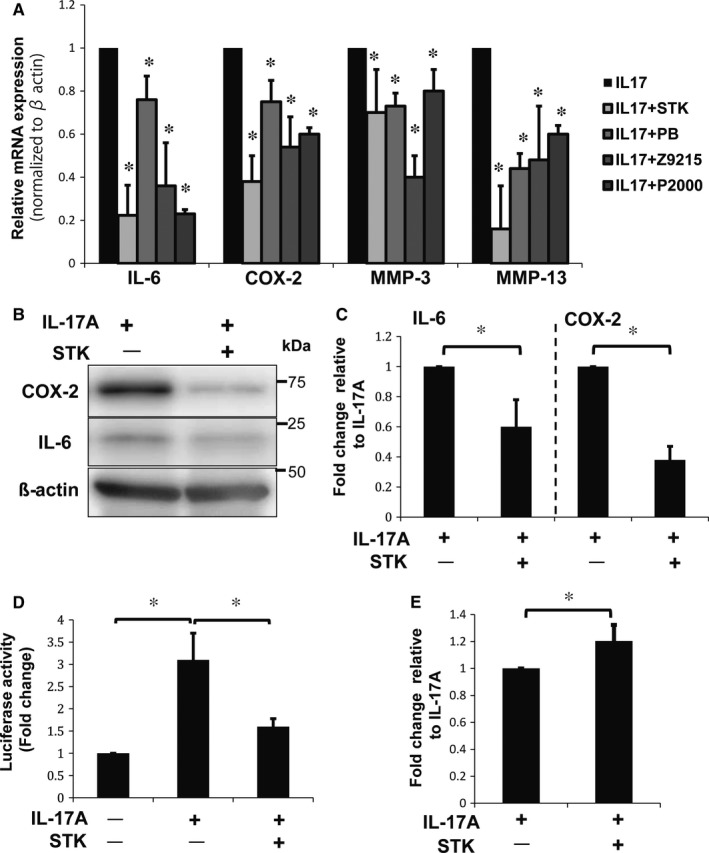
Effects of treatment with IL‐17A and small‐molecule inhibitors on NP cells. A, Real‐time PCR analysis of NP cells treated with STK630921 (STK), Z92151850 (Z9215), PB203263256 (PB) and P2000N‐53454 (P2000). The inhibitors significantly reduced expression of IL‐6, COX‐2, MMP‐3 and MMP‐13 mRNA. Results shown as mean ± SD; n = 3; *P <* *0.05. B, C, Western blot analysis of NP cells with STK treatment shows a reduction in IL‐6 and COX‐2 protein levels. Results shown as mean ± SD; n = 3; *P <* *0.05. D, COX‐2 promoter assay showing a loss of COX‐2 transcriptional activity in NP cells treated with STK. Results shown as mean ± SD; n = 3; *P <* *0.05. E, Alcian blue staining quantifying of NP cells treated with STK and IL‐17A. Optical density of the extracted dye was measured at 670 nm. Results shown as mean ± SD; n = 3, *P < 0.05

We evaluated the effectiveness of these four small‐molecule inhibitors, STK630921 (STK), Z92151850 (Z9215), PB203263256 (PB) and P2000N‐53454 (P2000) (Figure 4E), for blocking the IL‐17A–IL‐17RA interaction. NP cells were treated with 50 ng/mL of IL‐17A and 50 μg/mL of a small‐molecule inhibitor and incubated under hypoxic conditions for 24 hours. We found a significant decrease in IL‐6, COX‐2, MMP‐3 and MMP‐13 mRNA expression levels compared with cells treated with IL‐17A alone (Figure 5A). Of these small‐molecule inhibitors, STK had the highest GBVI/WSA_dG docking score, which represents the protein/ligand‐binding free energy^35^ (Figure 4E), so its effects were further evaluated. Both IL‐6 and COX‐2 protein levels decreased significantly in cells treated with both IL‐17A and STK, compared with cells treated with IL‐17A alone (Figure 5B,C). Similarly, the transcriptional activity of the COX‐2 promoter in cells treated with IL‐17A and STK was significantly decreased compared with that in cells treated with IL‐17A alone (Figure 5D). These results show the potential utility of small‐molecule inhibitors such as STK for down‐regulating the activity of IL‐17A and to control the activities of IL‐6, COX‐2 and MMPs. We evaluated the effects of STK on ECM of NP cells using alcian blue staining. The treatment of STK showed the significant effect on suppressing the degradation of sulphated glycosaminoglycans of NP cells compared with that in cells treated with IL‐17A alone (Figure 5E).

### 3.6 MAPKs regulate IL‐6 and COX‐2 expression after IL‐17A stimulation

**Figure 6:**
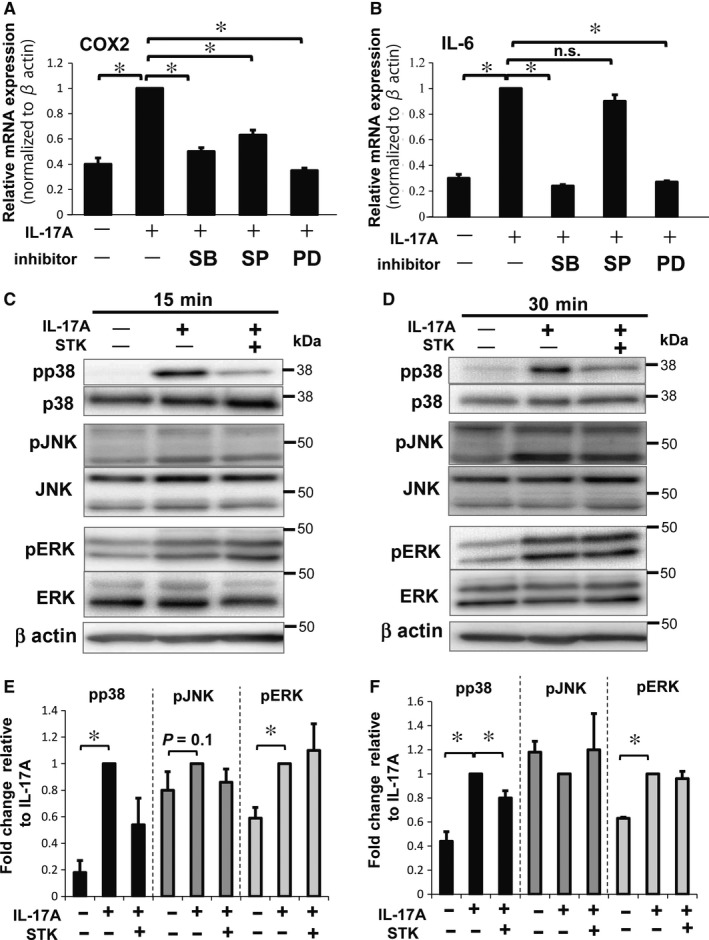
Evaluation of MAPK family members in NP cells treated with IL‐17A and small‐molecule inhibitors under hypoxia. A, B, Real‐time PCR analysis following treatment with IL‐17A and p38 inhibitor (SB203580, SB), JNK inhibitor (SP600125, SP) or ERK inhibitor (PD203580, PD). COX‐2 mRNA (A) was significantly decreased with all three inhibitors. IL‐6 mRNA (B) was significantly decreased in the cells with SB or PD treatment. Results shown as mean ± SD; n = 3, *P <* *0.05. C‐F, Western blotting and quantification of the phosphorylated forms of JNK, ERK, and p38 in NP cells treated with or without IL‐17A and STK at 15 minutes (C, E) and 30 minutes (D,F) of hypoxia. IL‐17A treatment significantly increased the phosphorylation of p38 and ERK at both 15 and 30 minutes compared with non‐treated NP cells. IL‐17A and STK treatments significantly decreased the phosphorylation of p38 compared with IL‐17A alone at 30 minutes (E, F). Results shown as mean ± SD; n = 3, *P <* *0.05

To clarify the pathophysiological action of IL‐17A and the role of inhibitors of the IL‐17A pathway in NP cells, we investigated the involvement of MAPKs in IL‐17A responses. First, we treated NP cells with pharmacological inhibitors of various MAPK pathway members (p38 inhibitor (SB203580), JNK inhibitor (SP600125), and ERK inhibitor (PD203580)) and assessed changes in COX‐2 and IL‐6 mRNA expression. NP cells were treated with 50 ng/mL of IL‐17A and 10 μmol/L of MAPK inhibitors for 24 hours under hypoxia. We found that all inhibitors significantly suppressed IL‐17A‐mediated induction of COX‐2 mRNA expression compared with cells treated with IL‐17A alone (Figure 6A). IL‐6 mRNA expression was significantly suppressed by SB and PD treatments, but SP treatment did not significantly suppress IL‐6 mRNA compared with cells treated with IL‐17A alone (Figure 6B). These results suggest that some expression of COX‐2 and IL‐6 may be regulated through at least one of the MAPKs.

Considering these results, we analysed the phosphorylation status of p38, JNK and ERK1/2 under IL‐17A treatment, with or without STK, using Western blotting. NP cells were treated with 50 ng/mL of IL‐17A and 50 μg/mL of STK under hypoxic conditions for 15 and 30 minutes. The phosphorylation of JNK tended to increase with IL‐17A stimulation at 15 minutes (Figure 6C,E), although there were no significant changes in phosphorylation under all conditions. Phosphorylation of p38 and ERK1/2 increased significantly with IL‐17A treatment compared with control at 15 and 30 minutes (Figure 6C‐F). Phosphorylated p38 tended to decrease at 15 minutes with IL‐17A and STK treatments and significantly decreased at 30 minutes, compared with treatment using IL‐17A alone (Figure 6E,F). In contrast, there was not a significant decrease in ERK1/2 phosphorylation with IL‐17A and STK treatments, compared with IL‐17A alone, at any of the time‐points tested (Figure 6E,F). These findings indicate that the IL‐17A inhibitor STK can modulate expression of IL‐6 or COX‐2 by suppressing p38 phosphorylation.

### 3.7 The small‐molecule inhibitor STK suppresses the effects of IL‐17A on human NP cells

**Figure 7:**
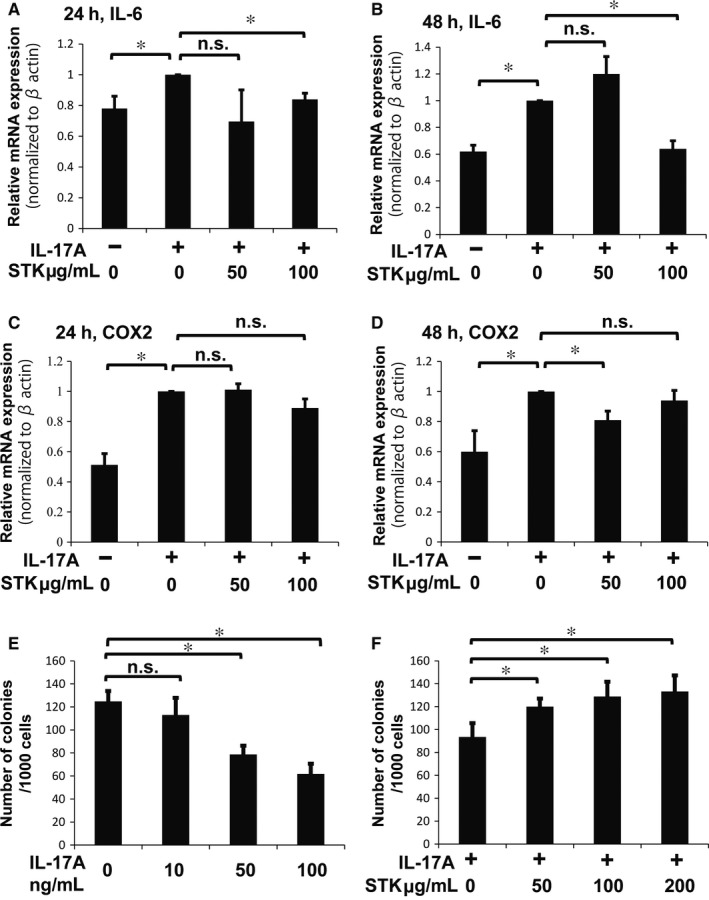
Effects of IL‐17A and STK treatments on human NP cells under hypoxia. A, B, IL‐6 mRNA expression in human NP cells treated with or without 50 ng/mL of IL‐17A and 50‐100 μg/mL STK for 24 hours (A) and 48 hours (B). Treatment with 100 μg/mL STK significantly suppressed expression of IL‐6 mRNA at both 24 and 48 hours. Results shown as mean ± SD; n = 4, *P <* *0.05. C, D, COX‐2 mRNA expression in human NP cells treated with or without 50 ng/mL of IL‐17A and 50‐100 μg/mL STK for 24 hours (C) and 48 hours (D). Treatment with 50 μg/mL STK significantly suppressed expression of COX‐2 mRNA at 48 hours (D). Results shown as mean ± SD; n = 4, *P <* *0.05. E, Quantification of colonies formed in NP cells treated with 0‐100 ng/mL IL‐17A after 10 days of culture in methylcellulose‐based medium. F, Quantification of colonies formed in NP cells treated with 50 ng/mL IL‐17Aand 0‐200 μg STK after 10 days of culture in methylcellulose‐based medium. Results shown as mean ± SD; n = 3, *P < 0.05

Human NP cells were treated with 50 ng/mL of IL‐17A, and 50 and 100 μg/mL of STK for 24 and 48 hours under hypoxia. IL‐6 and COX‐2 mRNA expression significantly increased after IL‐17A treatment compared with non‐treated cells (Figure 7A‐D). Further, 100 μg/mL of STK significantly decreased IL‐6 mRNA expression with IL‐17A stimulation compared with IL‐17A treatment alone; however, 50 μg/mL of STK did not result in any significant change at any time point (Figure 7A,B). Treatment with 50 μg/mL of STK did not significantly decrease expression of COX‐2 mRNA at 24 hours but decreased it significantly at 48 hours, compared with IL‐17A treatment alone (Figure 7C,D), although 100 μg/mL STK did not significantly change COX‐2 mRNA expression at any of the time‐points tested (Figure 7C,D). Furthermore, we analysed the colony‐forming assay in order to evaluate the effects of STK on ECM production in human NP cells. The IL‐17A treatment decreased the number of colonies, in a IL‐17A concentration‐dependent manner (Figure 7E). STK treatment significantly increased the number of IL‐17A colonies in all concentrations when compared to cells treated with 50 ng/mL IL‐17A alone (Figure 7F).These findings demonstrate that the small‐molecule inhibitor, STK, was effective in human NP cells.

## 4 DISCUSSION

In this study, we showed that IL‐17A signaling up‐regulated IL‐6, COX‐2, MMP‐3 and MMP‐13 expression in NP cells under hypoxia. Furthermore, IL‐17A affects the degradation of sulphated glycosaminoglycan, a major extracellular matrix(ECM) component produced by NP cells. In addition, these factors, the elevation of which characterizes IVD diseases,^7^, ^8^, ^9^, ^10^, ^11^, ^13^ were down‐regulated by suppressing IL‐17A activation with a neutralizing antibody or small‐molecule inhibitors. These findings are the first to reveal that the IL‐17A–IL‐17RA interaction is a possible pharmacotherapeutic target and that small‐molecule inhibitors of IL‐17A–IL‐17RA might be utilized for IVD disease therapy.

With respect to the relationship between IL‐6 and IL‐17A, some reports have indicated that IL‐6 is elevated in response to IL‐17A‐activated STAT3 in some tumour cells.^39^, ^40^ In our study, IL‐6 was increased by IL‐17A stimulation and down‐regulated when IL‐17A signalling was suppressed using an anti‐IL‐17A antibody or an IL‐17A inhibitor. These results suggest that IL‐17A may be one of the elements that controls expression of IL‐6 in NP cells. Previous studies have reported that IL‐6 is secreted by IVD cells in the absence of macrophages^41^ and that its expression is elevated in injured IVD cells or herniated discs.^31^, ^42^ Others have shown that IL‐6 down‐regulates disc matrix formation and promotes disc degeneration^7^, ^9^, ^28^, ^30^ and contributes to expression of inflammatory mediators such as TNF‐α and PGE2,^28^, ^30^ which lead to neuropathic pain.^29^, ^43^ We found that IL‐17A treatment increases IL‐6, COX‐2, MMP‐3 and MMP‐13 expression and that IL‐6 treatment alone also increases COX‐2, MMP‐3 and MMP‐13 expression. These findings suggest that IL‐17A up‐regulates IL‐6 expression, which in turn induces COX‐2 and MMPs in NP cells, contributing to the pathomechanism of disc degeneration and symptoms. Similarly, COX‐2 is an important enzyme in PGE‐2 biosynthesis in disc cells^8^, ^44^ that plays key roles in the pathophysiology of IVD disease, including lower back pain and sciatica.^7^, ^8^, ^17^ These studies indicate that COX‐2 may be a major pharmacotherapeutic target in IVD disease, in addition to IL‐6. In fact, selective COX‐2 inhibitors are used as painkillers in clinical medicine. We showed that COX‐2 mRNA and protein expression levels, and promoter activity, were elevated by IL‐17A stimulation and reduced by IL‐17A‐neutralizing antibody or inhibitors in NP cells. These findings demonstrate that IL‐17A regulates COX‐2 expression in NP cells, resulting in lower back pain and sciatica.

Moreover, we evaluated the influence of IL‐17A on the ECM deposition by NP cells. NP cells secrete and organize the ECM, which is composed of highly hydrated proteoglycans and collagen, and this ECM molecular composition plays a central role in the normal functioning of the IVD.^45^, ^46^ Degradation of the ECM accounts for degeneration of the IVD.^13^, ^46^ Our findings reveal that IL‐17A treatment decreased the deposition of sulphated glycosaminoglycans, major ECM component of NP cells.

Taken together, our findings suggest that IL‐17A is a promising pharmacotherapeutic target of small‐molecule inhibitors as an IVD therapeutic strategy. We evaluated the utility of small‐molecule inhibitors of the IL‐17A–IL‐17RA interaction and assessed their effects. There are some differences between small‐molecule inhibitors and neutralizing antibodies. The IL‐17A neutralizing antibody binds to the region of the IL‐17A protein, which is the immune‐specific antigen, and neutralizes the activity of IL‐17A, while small‐molecule inhibitors bind to the IL‐17A‐binding site region of the IL‐17 receptor on the cells. There was not an immune‐specific relationship between the small‐molecule inhibitor and IL‐17A. The four small‐molecule inhibitors we identified bound to the surface of IL‐17RA at the binding pocket region, blocking the IL‐17A–IL‐17RA interaction in NP cells. The small‐molecule inhibitors suppressed expression of IL‐6, COX‐2, MMP‐3, MMP‐13 and an IL‐17A‐neutralizing antibody in NP cells. The results of these studies suggest that small‐molecule inhibitors that bind the IL‐17A‐binding region of IL‐17RA are prospective anti‐inflammatory molecules that may block degeneration of IVDs, which may warrant the development of an IL‐17A inhibitor as a treatment option for patients with IVD disease.

We evaluated the effects of the STK small‐molecule inhibitor on the intracellular IL‐17A signalling pathway. A previous study reported that IL‐17A increased COX‐2 expression via the activation of MAPK pathways in NP cells under normoxic conditions.^32^ In our study, treatment with MAPK inhibitors showed that p38 and ERK inhibitors suppressed not only COX‐2 but also IL‐6 mRNA expression; however, the JNK inhibitor suppressed only COX‐2 expression. Phosphorylation of p38 and ERK increased significantly, and JNK phosphorylation tended to increase with IL‐17A treatment after 15‐30 minutes under hypoxic conditions. This indicates that p38 and ERK are important for regulation of IL‐6 and COX‐2 expression. The STK small‐molecule inhibitor was effective for suppressing p38 phosphorylation, suggesting that p38 coordinates both IL‐6 and COX‐2 regulation, and that STK particularly affects expression of IL‐6 or COX‐2 by suppressing p38 phosphorylation. Inhibition of p38 was reported to down‐regulate the induction of IL‐6 in NP cells^47^; this study and our results suggest the possibility that p38 plays an important role in inducing IL‐6 in NP cells. Although the small‐molecule inhibitor did not suppress phosphorylation of ERK, this does not suggest a disadvantage of IL‐17A inhibition. ERK activation is necessary for NP cell survival under hypoxic conditions,^48^ and thus excessive suppression of ERK by inhibitors is not beneficial to NP cells. Although many intracellular pathways are involved in the regulation of different factors, our findings suggest that the STK small‐molecule inhibitor mainly affects the p38 pathway and results in suppression of IL‐6 and COX‐2.

Further, we evaluated the effects of the STK small‐molecule inhibitor on recovery of sulphated glycosaminoglycan production, a major ECM component of NP in culture. STK showed its effectiveness to suppress the loss of ECM caused by IL‐17A stimulation. These results also demonstrate that STK small‐molecule inhibitor is useful to suppress degeneration of IVDs.

In addition, we verified the effects of the STK small‐molecule inhibitor in human NP cells. Although there are some differences in the conditions used for analysis of rat NP cells and human cells (eg, concentration of inhibitor and treatment period), the STK small‐molecule inhibitor also down‐regulated IL‐6 and COX‐2 expression in human NP cells. This emphasizes the utility of small‐molecule inhibitors for IL‐17A–IL‐17RA as a pharmacological therapy for IVD disease. Additionally, we analysed the effects of STK on human NP cell spheroid colony‐forming capability, which reflects their ability to self‐renew, as well as the ability to produce ECM.^45^, ^49^ The number of human NP cell spheroid colonies was decreased by treatment with IL‐17A and was further improved by STK small‐molecule inhibitor treatment. These findings demonstrate that the STK small‐molecule inhibitor can facilitate self‐renewal and ECM production, in response to the catabolic effects initiated by IL‐17A signalling and provide insights for new therapeutic option for IVD disease.

The major limitations of this study are described below. We used both rat NP cells and human NP cells, and the notochondal phenotypes are not identical, with the presence of a portion of notochondal cells in adult discs differing in rats and humans. Thus, there is the possibility that the IVD degeneration process in rats is not analogous to that in humans. Our observations using human NP cells included sufficient samples to be statistically analysed; however, it is difficult to collect IVDs from patients of all the same ages in order to have a more controlled patient group. Therefore, concern over age variation of our patients and the effect it has on the degeneration process of IVDs still remains.

In summary, the present study shows that IL‐17A increases expression of IL‐6, COX‐2, MMP‐3 and MMP‐13. Further, inhibition of IL‐17A binding to IL‐17RA suppressed IL‐17A signalling and reduced expression of these factors in NP cells under hypoxic conditions, and the small‐molecule inhibiters that bound to the IL‐17A–IL‐17RA‐binding region were effective in NP cells. Although further studies on the detailed mechanisms of action are required, our study offers insight into the potential utility of small‐molecule inhibiters of IL‐17A–IL‐17RA as a pharmacological therapy in IVD disease.

## CONFLICT OF INTEREST

The authors confirm that there are no conflicts of interest.

## AUTHOR CONTRIBUTIONS

KS, YN, EM and NH performed the research. KS, NH and DS analysed the data and wrote the manuscript. DS designed the research study. HT, NQ, OT, KS and MW contributed essential reagents or tools.
